# Deciphering the Potential Role of Symbiotic Plant Microbiome and Amino Acid Application on Growth Performance of Chickpea Under Field Conditions

**DOI:** 10.3389/fpls.2022.852851

**Published:** 2022-05-12

**Authors:** Munazza Rafique, Abid Ali, Muhammad Naveed, Tasawar Abbas, Asma A. Al-Huqail, Manzer H. Siddiqui, Ahmad Nawaz, Martin Brtnicky, Jiri Holatko, Antonin Kintl, Jiri Kucerik, Adnan Mustafa

**Affiliations:** ^1^Soil Bacteriology Section, Ayub Agricultural Research Institute, Faisalabad, Pakistan; ^2^Institute of Soil and Environmental Sciences, University of Agriculture, Faisalabad, Pakistan; ^3^Department of Botany and Microbiology, College of Science, King Saud University, Riyadh, Saudi Arabia; ^4^Department of Entomology, University of Agriculture, Faisalabad, Pakistan; ^5^Institute of Chemistry and Technology of Environmental Protection, Faculty of Chemistry, Brno University of Technology, Brno, Czechia; ^6^Department of Agrochemistry, Soil Science, Microbiology and Plant Nutrition, Faculty of AgriSciences, Mendel University in Brno, Brno, Czechia; ^7^Agricultural Research, Ltd., Troubsko, Czechia; ^8^Institute for Environmental Studies, Faculty of Science, Charles University in Prague, Prague, Czechia

**Keywords:** *Rhizobium*, PGPR, L-methionine, chickpea, plant-microbe interaction

## Abstract

The unprecedented rise in the human population has increased pressure on agriculture production. To enhance the production of crops, farmers mainly rely on the use of chemical fertilizers and pesticides, which have, undoubtedly, increased the production rate but at the cost of losing sustainability of the environment in the form of genetic erosion of indigenous varieties of crops and loss of fertile land. Therefore, farming practices need to upgrade toward the use of biological agents to maintain the sustainability of agriculture and the environment. In this context, using microbial inoculants and amino acids may present a more effective, safer, economical, and sustainable alternative means of realizing higher productivity of crops. Therefore, field experiments were performed on chickpea for two succeeding years using *Rhizobium* and L-methionine (at three levels, i.e., 5, 10, and 15 mg L^–1^) separately and in combinations. The results show that the application of *Rhizobium* and all the three levels of L-methionine increased the growth and yield of chickpea. There was a higher response to a lower dose of L-methionine, i.e., 5 mg L^–1^. It has been found that maximum grain yield (39.96 and 34.5% in the first and second years, respectively) of chickpea was obtained with the combined use of *Rhizobium* and L-methionine (5 mg L^–1^). This treatment was also the most effective in enhancing nodule number (91.6 and 58.19%), leghemoglobin (161.1 and 131.3%), and protein content (45.2 and 45%) of plants in both years. Likewise, photosynthetic pigments and seed chemical composition were significantly improved by *Rhizobium* inoculation. However, these effects were prominent when *Rhizobium* inoculation was accompanied by L-methionine. In conclusion, utilizing the potential of combined use of L-methionine and microbial inoculant could be a better approach for developing sustainable agriculture production.

## Introduction

The global population is predicted to reach 9.2 billion by 2050, with a projected increase in food demand. It seems necessary to increase agricultural production to feed the growing population. Current agricultural practices are aimed at maximizing the production of crops with scant care for the quality of produce, food safety, human health, and preservation of natural resources. Intensive reliance on agrochemicals has posed a challenge to the sustainability of the traditional farming system, as well as food security, protection of human health, and productivity of natural resources (Abbas et al., 2018). The most pressing concern is the loss of soil fertility, which reduces soil productivity. Therefore, employing natural organic and biological inputs, reversal of the decline in soil health may be possible. Legumes are well-known for developing and restoring soil fertility owing to their mutualistic relationship with symbiotic nitrogen-fixing bacteria. Chemical fertilizers are utilized to fulfill the nitrogen requirements of plants and attain higher yields of crops, but they cause negative ecological impacts (Tariq et al., 2020). Nitrogen-fixing rhizobia are inoculated in legume crops to gear up the natural process of biological nitrogen fixation to fulfill nitrogen requirements of crops and restore soil fertility (Naseem et al., 2018; Saeed et al., 2021). Effective nitrogen fixation symbiosis with rhizobia is key to the success of symbiosis relationships with legumes. However, various agricultural soils lack sufficient rhizobial populations. Also, naturally existing populations may be poor. It reduces their efficiency to fix nitrogen successfully, resulting in lower agricultural yield. Therefore, the use of rhizobial inoculants is an important means to combine its known significance in improving crop yield and biological control of plant diseases and pests (Yadav et al., 2021). These situations provide an opportunity to outline the use of symbiotic microorganisms to enrich the rhizosphere of legumes to promote effective nodules, nitrogen fixation, and the next prosperity of food production (Ali et al., 2017; Wolde-meskel et al., 2018; Matse et al., 2020). For example, rhizobial inoculants in legumes have been improving agricultural productivity and forming a familiar method since ancient times (Abdullahi et al., 2013), which provides an effective and appropriate method for legume rhizosphere and soil (Ahmad et al., 2019). This is vital for increasing crop yield in farmlands, especially where the supply of nitrogen fertilizers restricts crop production and productivity. The practice increases the infection establishment, nodulation, biomass, yield component, yield, and nutrient uptake of the legume crops (Khan et al., 2018).

The N is a critical element for legume crops because it is an integral component of chloroplast, phospholipids, and proteins (Nawaz et al., 2018). Uptake of nitrogen, its assimilation, and usage shows an essential part in improving the productivity of plants (Yuan et al., 2012). To offset the chemical-based agricultural industry, sustainable farming approaches are needed. From this perspective, exogenous use of amino acids is also considered to improve the productivity of crops. These are a type of phytostimulators (substances that enhance the growth of plants) that supply plant nutrients, which results in improved plant growth, yield, and commercial output (du Jardin, 2015; Rouphael et al., 2018). They are not only getting familiar with alleviating injuries of abiotic stresses to plants (Kowalczyk et al., 2008) but also serve as phytohormone precursors, i.e., tryptophan and methionine (Maeda and Dudareva, 2012; Calvo et al., 2014; Tatjana et al., 2015; Khan et al., 2019). Phytostimulators also regulate nitrogen uptake in plants (Miller et al., 2007), growth of roots (Walch-Liu and Forde, 2007; Calvo et al., 2014; Halpern et al., 2015; Weiland et al., 2016), and metabolism of antioxidants (Teixeira et al., 2017). Exogenous application of amino acids can promote root development, thereby increasing the ability of nitrogen fixation owing to an increase in root surface area for absorption of nutrients (Hildebrandt et al., 2015; Weiland et al., 2016). Direct use of amino acids or their products can change the absorption and assimilation of nitrogen, and various enzymes are involved in mediating this mechanism (Calvo et al., 2014; du Jardin, 2015). The latest research (Teixeira et al., 2017) found that the application of amino acids or their products through seeds or foliar treatment had different effects on legume crops. The use of amino acids alone as signal components improves antioxidant enzymes’ activities and results in nutrient absorption (Giose et al., 2012). Earlier studies have shown that L-methionine is used as an amino acid for the synthesis of growth regulators in plants (e.g., auxins, brassinosteroides, and cytokinins). Additionally, L-methionine is a precursor of multiple essential biomolecules such as vitamins, polyamines, cofactors (Paciorek et al., 2005; Yong et al., 2014), and antioxidants. However, the impact of single amino acids, particularly in seed applications, is poorly understood. In addition, most studies have used amino acid mixtures as well as other application strategies, such as soil treatment and foliar application (Sadak et al., 2015; Abd El-Aal, 2018; Khan et al., 2019). This study is based on the theory that the use of L-methionine alone and together with rhizobia can increase nitrogen absorption and other plant growth factors, and leads to increased productivity of chickpea. Therefore, the purpose of this research is to evaluate the effects of L-methionine alone and in combination with *Rhizobium* on the growth, physiology, and yield of chickpea crops.

## Results

### Biochemical Characterization

During microscopic studies, Gram-negative rod-shaped bacterial cells were observed. Colonies of isolates were circular, oval shape, 2–3.3 mm, cream yellow, milky white, cream white, translucent, and opaque, and some were raised while others were flattened (Table 1). All the isolates showed the ability to form biofilm and produce exopolysaccharides except for CPRH-4. Some of the isolates had the ability to solubilize inorganic phosphorus else than CPRH-2, CPRH-6, and CPRH-7. In this study, it was observed that all isolates grown with 0.5% (w/v) NaCl continued to grow with 2.5% (w/v) NaCl and could be grown with yeast extract with a pH range of 4- 8. In a prevailing research study, it was found that the most effective pH for the growth of rhizobia isolates is 6–7. The isolates grew slightly at pH 4 and pH 8. The potential of all the isolates to produce citric acid was tested; it was observed that some of the isolates (CPRH-1, CPRH-3, CPRH-4, CPRH-5, CPRH-7, CPRH-9, and CPRH-10) were positive for citric acid production, while the others were negative. During the urease test, the isolates CPRH-3 and CPRH-7 tested negative, while the remaining *Rhizobium* isolates showed positive results. All the isolates were streaked in bromothymol blue (BTB) and Congo red with Yeast Extract Mannitol Agar (YEMA) selective medium for further confirmation of *Rhizobium* characteristics. In the BTB test, all isolates grew within 48 h and changed the YEMA medium from blue to yellow, supporting its rapid growth and acid-producing properties. No isolate obtained a Congo red color in this test.

**TABLE 1:** Physiological, biochemical, and plant growth-promoting factors of chickpea nodule rhizobia.

Characteristics	Designated Isolates									
	CPRH-1	CPRH-2	CPRH-3	CPRH-4	CPRH-5	CPRH-6	CPRH-7	CPRH-8	CPRH-9	CPRH-10
Colony color	MW	CY	MW	CW	CY	CY	MW	CW	CY	MW
Colony shape	C	C	O	C	O	O	C	C	C	C
Colony Transparency	OP	T	T	T	OP	T	OP	OP	T	T
Colony Structure	F	R	R	R	F	R	F	F	R	R
Size (mm) Bacterium shape	2.3 Rod	2.4 Rod	2.1 Rod	3.0 Rod	3.0 Rod	2.0 Rod	2.2 Rod	2.6 Rod	2.4 Rod	3.3 Rod
Biofilm formation*^a,b^*	+	+	+	−	+	+	+	+	+	+
EPS production*^a,b^*	+	+	+	−	+	+	+	+	+	+
P-Solubilization potential*^a,b^*	+	−	+	+	+	−	−	+	+	+
Gram reaction	−	−	−	−	−	−	−	−	−	−
***Triple iron sugar test***										
Glucose	+	+	+	+	+	+	+	+	+	+
Sucrose	+	+	+	+	+	+	+	+	+	+
Maltose	+	+	+	+	+	+	+	+	+	+
***Biochemical Tests***										
Citrate test	+	−	+	+	+	−	+	−	+	+
Urease test	+	+	−	+	+	+	−	+	+	+
Starch Hydrolysis	+	+	−	+	+	−	+	+	+	+
Fluorescence assay	−	−	−	−	+	−	−	−	−	−
YEMA-CR test	NA	NA	NA	NA	NA	NA	NA	NA	NA	NA
YEMA-BTB test	^+^Y	^+^Y	^+^Y	^+^Y	^+^Y	^+^Y	^+^Y	^+^Y	^+^Y	^+^Y
***Growth conditions***										
***NaCl (%)***										
0.5	+	++	+	+ +	++	+	+ +	+	+ +	+ +‘
1.0	+ +	+	+ +	+	++	++‘	+ +	+	+ +	+ +‘
1.5	+	++	++‘	+ +	+	+ +	+ +‘	++	+	+ +
2.0	+	+	+	+	+	+	+	+	+	+ +
2.5	+	+	+	+	+	+	+	+	+	+
***pH***										
4	−	−	−	+	−	+	+	−	−	−
5	+	−	+	−	−	+	+	+	+	+
6	+	+	+	+	+	+	+	+	+	+
7	+	+	+	+	+	+	+	+	+	+
8	+	−	+	−	+	+	+	+	+	+
**Biolog identification and similarity index (%)**										
			76	78	73	77	76	80	78	82

*MW, milky white; CY, cream yellow; CW, cream white; C, circular; O, oval; OP, opaque; T, translucent; F, flattened; R, raised; a, Present; b, absent; NA, not absorbed; ^+^Y, yellow; (mean acid production) −, negative; +, positive; +, low efficiency; ++, high efficiency, a, absent; b+, present.*

The isolates have a strong ability to grow in the presence of different carbohydrate sources, such as glucose, sucrose, and maltose. All the rhizobia isolates used all carbohydrates as a carbon source. The starch hydrolysis test was positive for all strains except for CPRH-3 and CPRH-6. In addition, *Rhizobium* colonies, except for CPRH-5, did not fluoresce in King’s medium under ultraviolet light (Table 1).

All the isolates were similar to *Mesorhizobium ciceri* in Biolog identification. Nevertheless, in the case of other related CPRH-10 isolates, an extreme similarity index (82%) with *M. ciceri* was observed.

### Bacterial Growth and Indole Acetic Acid Production

All the isolates exhibit growth in yeast extract mannitol medium and have the potential to produce indole acetic acid (IAA) as presented in Table 2. The optical density (OD) of all isolates was higher than control. However, maximum bacterial growth was observed in CPRH-10. Similarly, in the case of IAA production, CPRH-10 has a maximum potential of IAA production (5.89 μg mL^–1^) in the mannitol medium of yeast extract amended with L-tryptophan (Table 2).

**TABLE 2:** Indole acetic acid (IAA) production with different isolates obtained from the nodules of *Cicer arietinum L*.

Isolates	Host plant	Growth OD (600 nm)	Production of IAA (μgmL^–1^)
Control	*C. arietinum L.*	1.20 ± 0.03	2.33 ± 0.12
CPRH-1	*C. arietinum L.*	1.35 ± 0.05	3.15 ± 0.14
CPRH-2	*C. arietinum L.*	1.56 ± 0.07	2.89 ± 0.22
CPRH-3	*C. arietinum L.*	1.46 ± 0.01	3.99 ± 0.23
CPRH-4	*C. arietinum L.*	1.26 ± 0.20	4.12 ± 0.16
CPRH-5	*C. arietinum L.*	1.56 ± 0.10	4.33 ± 0.17
CPRH-6	*C. arietinum L.*	1.28 ± 0.09	3.66 ± 0.11
CPRH-7	*C. arietinum L.*	1.38 ± 0.14	4.10 ± 0.18
CPRH-8	*C. arietinum L.*	1.57 ± 0.12	4.19 ± 0.10
CPRH-9	*C. arietinum L.*	1.44 ± 0.15	4.99 ± 0.12
CPRH-10	*C. arietinum L.*	1.78 ± 0.11	5.89 ± 0.15

### Growth and Symbiotic Efficiency Test

Effects of the different isolates on seed germination were observed. Later, ten *Rhizobium* isolates were investigated for their effects on the growth and symbiosis of chickpea in a greenhouse (Table 3). It is found that the *Rhizobium* isolates stimulated root and shoot growth and symbiotic efficiency (Table 3). In CPRH-10, seed germination (92.6%), seedling height (23.4 cm), and root length (15.9 cm) were recorded and were higher than those of the control. However, CPRH-8 has the highest shoot dry matter (0.294 g), and CPRH-3 has higher symbiosis efficiency (118.45%).

**TABLE 3:** Effect of *Rhizobium* inoculation on growth and symbiosis effectiveness in pot study.

Isolates	Germination (%)	Seedling height (cm)	Root length (cm)	shoot dry weight (g)	Percentage SE	Effectiveness
Control	81.20 ± 0.20	15.90 ± 0.10	11.40 ± 0.02	0.23 ± 0.04	−	−
CPRH-1	85.10 ± 0.11	17.80 ± 0.02	13.50 ± 0.10	0.24 ± 0.05	105.58	HE
CPRH-2	89.21 ± 0.52	19.40 ± 0.30	15.60 ± 0.05	0.22 ± 0.07	95.71	HE
CPRH-3	85.60 ± 0.14	20.20 ± 0.02	14.69 ± 0.08	0.27 ± 0.09	118.45	HE
CPRH-4	90.60 ± 0.56	18.20 ± 0.70	16.13 ± 0.01	0.24 ± 0.03	104.29	HE
CPRH-5	93.50 ± 0.33	21.20 ± 0.40	15.15 ± 0.13	0.20 ± 0.01	87.12	HE
CPRH-6	90.20 ± 0.25	24.20 ± 0.11	14.30 ± 0.16	0.23 ± 0.005	101.29	HE
CPRH-7	88.70 ± 0.34	18.40 ± 0.15	13.50 ± 0.06	0.18 ± 0.008	77.25	E
CPRH-8	86.50 ± 0.22	17.60 ± 0.18	14.78 ± 0.03	0.29 ± 0.004	126.18	HE
CPRH-9	87.56 ± 0.41	16.50 ± 0.20	13.89 ± 0.09	0.21 ± 0.006	92.27	HE
CPRH-10	92.60 ± 0.32	23.40 ± 0.10	15.90 ± 0.15	0.26 ± 0.009	115.02	HE

*SE, symbiotic efficiency; E, effective; HE, highly effective.*

### Agronomic Traits Under Field Conditions

The *Rhizobium* sp. strain CPRH-10 and L-methionine significantly improved the growth and yield of chickpea. It has been observed that the sole response of CPRH-10 and L-methionine was significant, and that this effect became more pronounced when used in combination. The highest grain yield (1,534 and 1,641 kg ha^–1^) in both years (I, II) was recorded in CPRH-10 + 5mg L^–1^ L-met application (Table 4) respectively. Similarly, dry matter yield and pod yield were also affected by the application of CPRH-10 and L-met. Maximum dry matter (6,000 and 6,293 kg ha^–1^) and pod yield (4,200 and 3,360 kg ha^–1^) were observed in CPRH-10 + 5mg L^–1^ L-met during years I and II, respectively (Table 4).

**TABLE 4:** Chickpea yield and biomass traits at different L-methionine levels and seed inoculation with *Rhizobium*.

Treatments	Variables							
	Grain Yield (kgha^–1^)		Dry matter yield (kgha^–1^)		Pod yield (kgha^–1^)		Plant height (cm)	
	I	II	I	II	I	II	I	II
C	1096.00 ± 42.3d	1220.00 ± 11.5g	4573.33 ± 208.2d	5466.67 ± 78.2f	3066.67 ± 120.3e	2453.33 ± 57.0e	60.00 ± 0.58e	64.70 ± 0.33e
*Rhiz*	1274.00 ± 14.5 bc	1370.00 ± 23.1e	4993.33 ± 202.1cd	5626.67 ± 70.6ef	3500.00 ± 152.1d	2800.00 ± 40.6cd	67.00 ± 1.16de	66.00 ± 1.00e
Met (5)	1309.00 ± 54.3b	1441.00 ± 16.6c	5300.00 ± 369.9bcd	5866.67 ± 96.2cd	3666.67 ± 33.5cd	2933.33 ± 26.7c	69.00 ± 0.51de	67.00 ± 1.14de
Met (10)	1260.00 ± 32.2bc	1403.00 ± 6.6d	5526.67 ± 196.6abc	5706.67 ± 67.4de	3400.00 ± 100.1de	2720.00 ± 52.8d	69.90 ± 0.36cd	69.60 ± 0.59cd
Met (15)	1158.00 ± 30.4cd	1315.00 ± 15.2f	5400.00 ± 115.4bc	5946.67 ± 63.3c	3500.00 ± 57.4d	2800.00 ± 46.2cd	72.00 ± 0.33bc	71.00 ± 0.50bc
*Rhiz* + Met (5)	1534.00 ± 24.1a	1641.00 ± 38.4a	6300.00 ± 251.0a	6640.00 ± 23.1a	4400.00 ± 116.4a	3520.00 ± 92.3a	76.00 ± 1.20a	75.30 ± 0.33a
*Rhiz* + Met (10)	1490.00 ± 55.4 a	1540.00 ± 26.4b	6000.00 ± 57.9ab	6293.33 ± 26.7b	4200.00 ± 123.5ab	3360.00 ± 30.5ab	74.00 ± 1.12ab	73.30 ± 1.70ab
*Rhiz* + Met (15)	1356.00 ± 30.7 b	1515.00 ± 10.1b	5673.33 ± 136.8abc	6480.00 ± 70.4a	4000.00 ± 50.8b	3200.00 ± 43.2b	73.00 ± 1.73abc	72.70 ± 1.20abc
LSD	120.710	28.210	153.230	176.612	340.530	164.031	3.402	3.302

*Control (C), Rhizobium (CPRH-10) inoculation (Rhiz), L-methionine 5 mg L^–1^ [Met (5)], L-methionine 10 mg L^–1^ [Met (10)], and L-methionine 15 mg L^–1^ [Met (15)]. Means followed by equal do not differ by LSD test, at 5% probability.*

It was observed that rhizobial inoculation gave a significantly positive response to plant height. However, the CPRH-10 along with L-met (5 mg L^–1^) gave a maximum increase in plant height (76 cm in year I and 75.3 cm in year II) (Table 4). The application of *Rhizobium* and L-methionine significantly enhanced the emergence percentage of chickpea plants (Table 5). The highest emergence percentage (91.0 and 82.0% in year I and year II, respectively) was obtained in treatment where seed inoculation with CPRH-10 along with 5 mg L^−1^ L-methionine was done as compared to control treatment (Table 5). The results showed significant effects on maturity time with the use of the inoculation technique and amino acid. The maturity time was the longest in non-inoculation (Table 5). A steady decrease in maturity days was observed in *Rhizobium* inoculation along with 5mg L^–1^ L-methionine treatment in both years (106 and 98.89 days, respectively).

**TABLE 5:** Chickpea growth and nodulation traits at different L-methionine levels and seed inoculation with *Rhizobium*.

Treatments	Variables									
	Emergence (%)		Maturity time (days)		Number of nodules plant^–1^		Nodular volume (cm^3^)		Dry nodules weight (g plant^–1^)	
	I	II	I	II	I	II	I	II	I	II
C	72.00 ± 0.58f	67.00 ± 0.51e	118.00 ± 0.58a	109.70 ± 0.54a	16.70 ± 0.67e	17.70 ± 0.33e	0.58 ± 0.05e	0.65 ± 0.012e	0.14 ± 0.005f	0.16 ± 0.04e
*Rhiz*	80.00 ± 0.38d	72.00 ± 1.08d	114.00 ± 0.77b	106.00 ± 0.28b	23.30 ± 0.88c	22.90 ± 0.75c	0.82 ± 0.02c	0.85 ± 0.032c	0.32 ± 0.06c	0.35 ± 0.07b
Met (5)	83.00 ± 0.34c	75.00 ± 0.28c	113.10 ± 1.12bc	105.10 ± 0.22bc	21.00 ± 0.58cd	20.30 ± 0.24d	0.74 ± 0.03cd	0.75 ± 0.03d	0.30 ± 0.01cd	0.27 ± 0.03c
Met (10)	79.00 ± 1.12d	71.00 ± 1.04d	116.20 ± 0.88ab	107.60 ± 0.82ab	19.30 ± 0.67d	19.70 ± 0.18d	0.68 ± 0.006d	0.73 ± 0.05de	0.28 ± 0.03e	0.26 ± 0.05c
Met (15)	76.00 ± 0.23e	68.00 ± 0.33e	111.30 ± 0.89cd	103.20 ± 0.93cd	20.00 ± 0.25d	19.00 ± 0.11de	0.70 ± 0.001d	0.70 ± 0.021de	0.29 ± 0.02de	0.24 ± 0.14d
*Rhiz* + Met (5)	91.00 ± 0.45a	82.00 ± 0.79a	106.00 ± 1.00e	98.90 ± 1.12e	32.00 ± 1.02a	28.00 ± 0.21a	1.12 ± 0.009a	1.04 ± 0.024a	0.45 ± 0.07a	0.36 ± 0.05a
*Rhiz* + Met (10)	89.00 ± 0.12b	80.00 ± 1.01b	107.10 ± 0.67e	99.20 ± 0.62e	30.00 ± 0.74ab	25.70 ± 0.15b	1.05 ± 0.1ab	0.95 ± 0.034b	0.44 ± 0.02a	0.34 ± 0.02b
*Rhiz* + Met (15)	87.00 ± 0.47b	78.00 ± 0.90b	108.20 ± 0.33de	100.80 ± 0.31de	28.30 ± 0.80b	24.00 ± 0.10bc	0.99 ± 0.02b	0.89 ± 0.011bc	0.40 ± 0.08b	0.33 ± 0.01c
LSD	2.491	2.242	2.745	2.551	2.502	1.847	0.087	0.073	0.022	0.021

*Control (C), Rhizobium (CPRH-10) inoculation (Rhiz), L-methionine 5 mg L^–1^ [Met (5)], L-methionine 10 mg L^–1^ [Met (10)], and L-methionine 15 mg L^–1^ [Met (15)]. Means followed by equal do not differ by LSD test, at 5% probability.*

As shown in Table 5, inoculation with the rhizobial isolates significantly (*P* ≤ 0.05) affects a number of nodules plant^–1^. Separate CPRH-10 inoculation increased the number of nodules (23.3 and 22.9) in both years and further increased using amino acids. The highest number of nodules plant^–1^in both years (32 and 28, respectively) was obtained by the combined application of CPRH-10 and L-met (5 mg L^–1^).

Rhizobial inoculation significantly improves nodule volume (Table 5). The highest nodule volume (1.12 and 1.04 cm^3^plant^–1^) was obtained from CPRH-10 inoculation + L-methionine (5 mg L^–1^) in years I and II. It has been observed that nodule dry weight positively responded with the inoculation of *Rhizobium*. An increase in nodule dry weight was obtained by sole rhizobial inoculation (0.32 and 0.34 g plant^–1^) and was further increased by *Rhizobium* inoculation and L-methionine (5 mg L^–1^), i.e., 0.45 and 0.36 g plant^–1^ in years I and II, respectively. This result revealed that minimum nodule dry weight was recorded when no treatment was applied.

**FIGURE 1:**
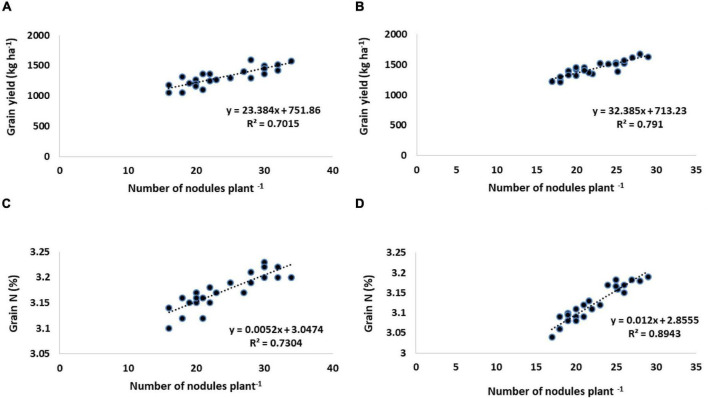
Relationship between number of nodules plant^–1^ with grain yield (kg ha^–1^) and grain nitrogen contents (%) of chickpea. **(A,C)** Year I. **(B,D)** Year II.

Rhizobial inoculation as well as its combined application with amino acids proved a significant potential in improving the number of pods, the weight of 100 seeds, and harvest index (Table 6). Without inoculation control, the smallest number of pods recorded was 24.94 (first year) and 19.07 (second year). CPRH-10 + L-met (5 mg L^–1^) gave the highest number of pods plant^–1^. During these 2 years, the number of pods plant^–1^increased by 8% compared with *Rhizobium* alone and 21–36% compared with the untreated control. The control has the lowest average weight of 100 seeds (18.5 and 15.55 g), but single inoculation with rhizobia significantly increases the weight of 100 seeds, which is an increase of 16–20% compared to the uninoculated control. The largest increase in 100-seed weight by 24.13 g in the first year and 19.4 g in the second year compared to the control was caused by inoculation with CPRH-10 and the application of L-methionine at a rate of 5 mg L^–1^. Current research shows that the number of nodule plants^–1^ is positively correlated with the yield of chickpeas and nitrogen concentration in the grains (Figure 1).

**TABLE 6:** Chickpea reproductive and biochemical traits at different L-methionine levels and seed inoculation with *Rhizobium*.

Treatments	Variables									
	Number of pods plant^–1^		100 seed weight (g)		Harvest index (%)		Leghemoglobin (mg g^–1^)		Seed Protein (%)	
	I	II	I	II	I	II	I	II	I	II
C	24.94 ± 0.95c	19.07 ± 0.37d	18.50 ± 0.80e	15.55 ± 0.64d	23.99 ± 0.59ab	22.33 ± 0.42c	0.18 ± 0.009g	0.16 ± 0.003f	15.70 ± 0.67f	14.57 ± 0.31f
*Rhiz*	27.83 ± 0.12abc	23.86 ± 0.98bc	21.49 ± 0.75bcd	18.65 ± 0.23abc	25.60 ± 1.19a	24.35 ± 0.30a	0.28 ± 0.005d	0.23 ± 0.012d	19.70 ± 0.33cd	18.29 ± 0.62cd
Met (5)	28.12 ± 0.81abc	22.29 ± 0.29c	20.60 ± 0.31de	17.76 ± 0.76bc	25.19 ± 0.55a	24.59 ± 0.52a	0.25 ± 0.003e	0.21 ± 0.004de	18.50 ± 0.29de	17.24 ± 0.82de
Met (10)	28.82 ± 0.44ab	22.37 ± 0.37c	21.80 ± 0.42bcd	17.67 ± 0.33c	22.84 ± 0.80ab	24.60 ± 0.32a	0.22 ± 0.015ef	0.19 ± 0.006e	17.70 ± 0.88e	16.43 ± 0.27e
Met (15)	26.28 ± 0.65bc	22.37 ± 0.23c	21.02 ± 0.83cd	17.57 ± 0.78c	21.44 ± 0.50b	22.12 ± 0.24c	0.20 ± 0.002f	0.20 ± 0.007de	18.40 ± 0.19de	17.1 ± 0.17de
*Rhiz* + Met (5)	30.16 ± 0.60a	25.88 ± 0.48a	24.13 ± 0.48a	19.40 ± 0.42ab	24.40 ± 0.82ab	24.73 ± 0.37a	0.47 ± 0.012a	0.37 ± 0.008a	22.80 ± 0.23a	21.17 ± 0.22a
*Rhiz* + Met (10)	29.02 ± 1.00ab	24.51 ± 0.55ab	23.22 ± 0.93ab	19.74 ± 0.79a	24.82 ± 0.70ab	24.47 ± 0.22a	0.42 ± 0.010b	0.33 ± 0.012b	21.70 ± 0.41ab	20.15 ± 0.56ab
*Rhiz* + Met (15)	28.98 ± 0.58ab	23.61 ± 0.51bc	23.19 ± 0.46abc	19.71 ± 0.39a	23.89 ± 0.22ab	23.38 ± 0.19b	0.38 ± 0.007c	0.28 ± 0.100c	21.20 ± 0.60bc	19.685 ± 0.38bc
LSD	3.1251	1.6007	2.1717	1.6938	3.5917	0.8656	0.0214	0.0276	1.5660	1.4567

*Control (C), Rhizobium (CPRH-10) inoculation (Rhiz), L-methionine 5 mg L^–1^ [Met (5)], L-methionine 10 mg L^–1^ [Met (10)], and L-methionine 15 mg L^–1^ [Met (15)]. Means followed by equal do not differ by LSD test, at 5% probability.*

### Chemical Analysis

**FIGURE 2:**
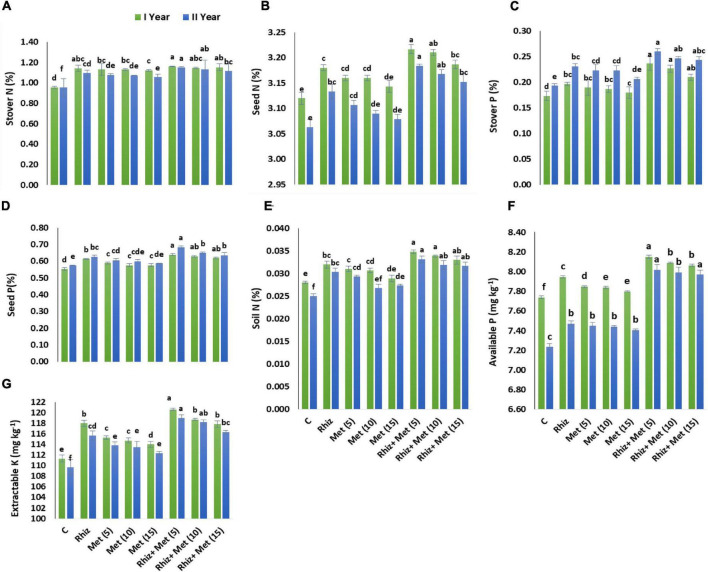
Effect of seed inoculation and L-methionine application on nutrient contents. *Y*-axis bar shows standard error. **(A)** Stover N (%), **(B)** Seed N (%), **(C)** Stover P (%), **(D)** Seed P (%), **(E)** Soil N (%), **(F)** Available P (mg kg^−1^), **(G)** Extractable K (mg kg^−1^). Treatment abbreviations: control (C), *Rhizobium* (CPRH-10) inoculation (*Rhiz*), L-methionine 5 mg L^–1^ [Met (5)], L-methionine 10 mg L^–1^ [Met (10)], and L-methionine 15 mg L^–1^ [Met (15)].

The soil NPK content was significantly improved using *Rhizobium*, L-methionine, and their combinations (Figure 2). When applied separately, both L-methionine and rhizobia seed treatments increased soil nutrient content, but the largest increase in N, P, and K content (32, 10.77, 8.35% and 25, 5.29, and 9.17%, respectively) in years I and II were obtained by treating the plants together with the application of CPRH-10 and L-met (5 mg L^–1^) in two seasons.

In the case of plant nutrient content, the combined application of rhizobia and L- methionine (5 mg L^–1^) achieved higher StoverN (20.83% in the first year and 21.05% in the second year) and StoverP contents (41.17% in the first year and 36.84% in the second year). Similarly, rhizobia together with L-methionine (5mg L^–1^) increased the N and P contents of seeds by 3.2 and 3.9%, and 16.36 and 19.29%, respectively, compared with the control (Figure 2).

### Physiological Traits

The results indicated that leghemoglobin content in the chickpea root nodules and seed protein contents were increased with the treatment of *Rhizobium* and amino acid alone and together (Table 6). It was observed that a maximum increase in leghemoglobin content was recorded with the combined application of *Rhizobium* and L-methionine (5 mg L^–1^). It was 161.1 and 131.3% higher than that of the control in years I and II, respectively (Table 6). Significant differences in protein content were observed because of the application of *Rhizobium* and 3 levels of amino acid individually and in combinations (Table 6). The percentage increase in protein content by the combined application of *Rhizobium* and L-methionine (5 mg L^–1^) reached 45.2 and 45.5% compared to the control in years I and II, respectively. Using *Rhizobium*, L-met, and their combinations positively affected chlorophyll *a*, chlorophyll *b*, carotenoids, and total pigments in chickpea (Table 7). The largest increase in these contents was recorded in the treatment with CPRH-10 and L-methionine (5 mg L^–1^). Compared with the control, chlorophyll *a* was increased by 58.1 and 51.40%, chlorophyll *b* was increased by 57.1 and 92.3%, carotenoids were increased by 84.2 and 92.9%, and total chlorophyll was increased by 62 and 65% in the first and second years, respectively. In both seasons, a seed treatment with L-met improved all photosynthetic pigments. However, a better response was observed with amino acid and *Rhizobium* treatment.

**TABLE 7:** Chickpea physiological traits at different L-methionine levels and seed inoculation with *Rhizobium*.

Treatments	Variables							
	Chlorophyll *a* (mg g^–1^)		Chlorophyll *b* (mg g^–1^)		Carotenoids (mg g^–1^)		Total pigments (mg g^–1^)	
	I	II	I	II	I	II	I	II
C	0.93 ± 0.02f	1.05 ± 0.025g	0.56 ± 0.005f	0.39 ± 0.028e	0.19 ± 0.009f	0.14 ± 0.034d	1.68 ± 0.023g	1.58 ± 0.023h
*Rhiz*	1.09 ± 0.04d	1.23 ± 0.012f	0.65 ± 0.011d	0.57 ± 0.002c	0.26 ± 0.003d	0.19 ± 0.005c	2.01 ± 0.019d	1.99 ± 0.026d
Met (5)	1.02 ± 0.07e	1.15 ± 0.013d	0.61 ± 0.023e	0.50 ± 0.009d	0.24 ± 0.005e	0.17 ± 0.009c	1.87 ± 0.012e	1.82 ± 0.012e
Met (10)	1.00 ± 0.08e	1.14 ± 0.016e	0.60 ± 0.032e	0.48 ± 0.0006d	0.24 ± 0.007e	0.17 ± 0.006c	1.84 ± 0.09e	1.78 ± 0.020f
Met (15)	0.97 ± 0.09e	1.12 ± 0.008ef	0.58 ± 0.017ef	0.46 ± 0.007d	0.23 ± 0.004e	0.16 ± 0.026c	1.78 ± 0.017f	1.74 ± 0.019g
*Rhiz* + Met (5)	1.47 ± 0.03a	1.59 ± 0.024a	0.88 ± 0.046a	0.75 ± 0.01a	0.35 ± 0.011a	0.27 ± 0.016a	2.70 ± 0.06a	2.60 ± 0.029a
*Rhiz* + Met (10)	1.39 ± 0.06b	1.45 ± 0.030b	0.83 ± 0.006b	0.64 ± 0.026b	0.33 ± 0.002b	0.23 ± 0.020b	2.55 ± 0.07b	2.33 ± 0.017b
*Rhiz* + Met (15)	1.29 ± 0.10c	1.32 ± 0.023c	0.77 ± 0.020c	0.60 ± 0.020bc	0.31 ± 0.006c	0.22 ± 0.012b	2.37 ± 0.03c	2.14 ± 0.006c
LSD	0.0658	0.0754	0.0368	0.0575	0.0172	0.0201	0.050	0.0234

*Control (C), Rhizobium (CPRH-10) inoculation (Rhiz), L-methionine 5 mg L^–1^ [Met (5)], L-methionine 10 mg L^–1^ [Met (10)], L-methionine 15 mg L^–1^ [Met (15)]. Means followed by equal do not differ by LSD test, at 5% of probability.*

**FIGURE 3:**
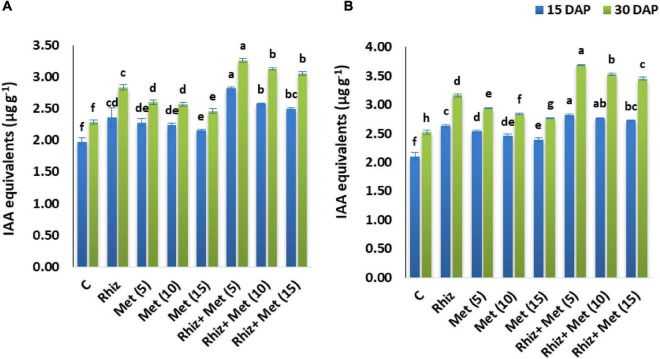
Effect of seed inoculation and L-methionine application on indole acetic acid (IAA) production. *Y*-axis bar shows standard error. Treatment abbreviations: control (C), *Rhizobium* (CPRH-10) inoculation (*Rhiz*), L-methionine 5 mg L^–1^ [Met (5)], L-methionine 10 mg L^–1^ [Met (10)], and L-methionine 15 mg L^–1^ [Met (15)]. **(A)** Year I. **(B)** Year II.

The IAA contents were determined after 15 and 30 Diammonium phosphate (DAP). It was found that a significant increase was recorded in both seasons in IAA contents compared to control (Figure 3). The highest IAA contents after 15 and 30 DAP were recorded by the combined application of *Rhizobium* and L-methionine (5 mg L^–1^), i.e., 2.82 and 3.27 μg g^–1^ in the season I and 2.79, 3.69 μg g^–1^ in season II.

## Discussion

Yearly biological nitrogen fixation generates 20–200 kg nitrogen per hectare in plant’s available forms in farming areas (Herridge et al., 2008) and maintains global food security. Among crops, most legumes are recognized because of their symbionts in root nodules with biological nitrogen fixation capabilities. *Rhizobium*-legume symbiosis has been investigated for decades as a reciprocal association. In this study, different rhizobial isolates were screened for plant growth promotion traits (Table 1). According to reports, the growth of rhizobium in the YEMA medium confirmed the morphological characteristics of *Rhizobium* (Somasegaran and Hoben, 1994). During culture preparation, Gram-negative and Congo red dye with non-absorbing behavior are typical characteristics of *Rhizobium vulgaris* (Abere et al., 2009). Studies have shown that a change in the color of the YEMA-BTB medium from blue to yellow indicates acid production and fast growth (Jida and Assefa, 2011). The diameter of colonies with oval and round edges is between 2 and 3.3 mm, which is consistent with other studies (Odee et al., 1997; Alemayehu, 2006). The biofilms and extracellular polysaccharides (EPSs) produced by the isolates may be universal components that protect bacteria from different biological and abiotic infestations in the soil (Khan et al., 2021). Rhizobia capable of tolerating environmental pressures may be more suitable for commercial inoculants (Saeed et al., 2021). These preliminary tests have previously been conducted to distinguish impurities in pure isolates of Rhizobia (Vincent, 1970). *Rhizobium* isolates were tested for IAA production in this study (Table 2). It is reported that the IAA produced by rhizobia plays a vital role in plant growth and interaction between Rhizobia and legumes. Production of plant hormones in nodules and their transport to the host through nodule symbionts was reported by Hunter (1989). Production of IAA from root nodule tryptophan by Rhizobia bacteria and its effect on nodule formation has been well-reported (Datta and Basu, 2000; Ghosh and Basu, 2006; Ghosh et al., 2013).

*Rhizobium* isolates were cultured in yeast extract mannitol (YEM) to test the potential for pH tolerance; they were grown in YEMA (Table 1). pH was maintained at 4–8, and 6–7 pH was found optimum for rhizobial growth. Nominal growth of the isolates was exhibited at pH 8 (Table 1). Deora and Singal (2010) reported that minimal variation in the pH of the medium might have a huge effect on the growth of nodule bacteria. Sensitivity of the Rhizobial isolates to high and low pH is observed commonly. It might be due to some strains of *Rhizobium* that have acid sensitivity, which has an important role in the regulation of internal pH and possibly accounts for their poor growth in cultures that have acidic pH (Riccillo et al., 2000).

All the isolates in this study were grown at different salt concentrations (Table 1), but it was found that *Rhizobium* has less ability to grow with the increase in salt concentration. It has been reported that extreme saline conditions suppress the proliferation and growth of *Rhizobium.* It has been speculated that some strains can stand and survive high salinity (Zahran, 1999; Keneni et al., 2010).

The most traditional microbes used as inoculants are “rhizobia.” They colonize the rhizosphere and form a symbiotic relationship with beans. They are employed to promote the productivity of plants by biological fixation of nitrogen; higher availability of plant nutrients by mobilization and dissolution, siderophores production, and release of plant growth hormones (Makoi et al., 2013; Koskey et al., 2021; Yadav et al., 2021). The present study showed a substantial role of *Rhizobium* inoculation on seedling emergence, as well as root and shoot elongation in pot study, as compared to untreated control (Table 3). This may be due to the inoculation by rhizobia of chickpea seeds by symbiotic nitrogen fixation to increase nitrogen absorption, which ultimately led to increased seedling height and root length. This result is similar to that of Farfour (2013). It reflects that rhizobial stain can improve the plant height of faba beans. Similarly, an increase in shoot and root length by bacterial strains was reported by El-Azeem et al. (2007). The results of the symbiosis effect (Table 3) show that the application of all 10 chickpea rhizobia in the study area is very effective. This result is similar to that of Gebremariam and Assefa (2018), which confirms that the symbiotic effect of *Rhizobium* strains isolated from faba beans is very effective.

A more multifarious amino acid cycle is crucial for symbiotic nitrogen fixation by *Rhizobium* in legumes. The use of amino acids in exogenous applications is getting more popular owing to their role in sustainable agriculture (Teixeira et al., 2017). By optimization of amino acid concentration, changes can be brought among various morphogenetic traits of crops; as higher concentrations mostly inhibitory role for growth in *Cicer arietinum* (John and Guha-Mukherjee, 1997). Various studies have shown that the optimal level of different amino acids depends on genotypes or species-specific characteristics. It should be determined before the recommended application (El-sharabasy et al., 2015). Gradually, plant ecologists are more interested in the role of amino acids in the nutritional life and productivity of plants (Rosati et al., 2000). We also found in field studies that when L-methionine was applied at a low concentration, the growth and yield attributes of chickpeas were higher compared with high concentrations (Table 4). Since L-methionine is also a precursor of ethylene, it is designated as a multifunctional plant hormone regulating plant growth and senescence. It has been observed that it is more beneficial at lower doses and has an inhibitory effect at higher doses (Khan, 2005; Khan et al., 2008).

In this research, it was found that inoculation with rhizobia could significantly promote the growth of chickpea sprouts and increase the number of pods and biomass. At the same time, inoculation with rhizobia can significantly affect the reproductive growth of chickpeas and increase the yield of pods and grains. This may be because inoculation with rhizobia can increase nitrogen content, promote the vegetative growth of plants, mainly root growth. Similar findings have also been reported in other studies in which chickpeas inoculated with rhizobia promoted plant growth, dry matter yield, pod number, grain yield, and nitrogen fixation under various climatic conditions. This is because of the extensive root system, nodulation ability, and strong and tall stems (Singh et al., 2003; Kasole et al., 2005). Our research shows that inoculation with rhizobia and L-methionine (5 mg L^–1^) has a greater significant effect on pod number, plant height, biomass, and grain yield. It can be anticipated that L-met, L-tryptophan, and L-glycine not only provide plants with nutrients but also transmit signals (Teixeira et al., 2017). Smaller doses are enough to improve plants, and these molecules can be used as signals for better physiology of plants. Previous studies have shown that spraying amino acids on plants in the form of foliar spray is an encouraging technique (Sadak et al., 2014). In this way, L-methionine attracts plants to absorb sulfur and nitrogen more significantly depending on application rate (Price, 2012; Vincill et al., 2012; Forde and Roberts, 2014; Santi et al., 2017).

This study has noted that *Rhizobium* inoculation + L- met has a greater positive impact on seed emergence, plant height, and earliest harvest than the untreated control (Tables 4, 5). The reason behind this is that increase in seed germination percentage and crop maturity is considered typical a phytohormonal response. Initially, the inoculated plants confirmed better emergence, which is probably due to the production of plant growth hormones, as phytohormones affect seed germination and, eventually, crop maturity. The potential of microorganisms in promoting plant germination and improving development has been adjusted to the *in vitro* and *in vivo* conditions of some crops and ornamental plants. It has been recognized that the promotion of seedling growth with rhizobia can produce biologically active compounds. It has been reported that plant growth stimulants can be used for better germination and production of plants (Tsavkelova et al., 2007; Nazli et al., 2020; Rafique et al., 2021).

Inoculation with rhizobia under field conditions can cause significant differences in nodule count, dry weight, and volume (*p* ≤ 0.05) (Table 6). Changes in the number of root nodule plants-1 and dry weight of nodules plants^–1^ indicate that the response of chickpea crops varies with rhizobia strains. Hence, this study agrees with a previous study. Jida and Assefa (2012) reported that faba bean produced a higher yield when a suitable symbiotic partner was provided. It has been found that rhizobia inoculation resulted in significant improvement in the dry weight of chickpea nodules (Table 5). Voisin et al. (2003) showed that an increase in the dry weight of tuberculosis is related to the higher efficiency of symbiotic relationships during tuberculosis development. Graham et al. (2004) reported that the dry weight of tuberculosis was a significant indicator of symbiosis efficiency. Efficient rhizobia isolates stimulate nodule formation. They give a higher fixation of N and biomass to crops. *Rhizobium* inoculation can improve nodulation and nitrogen fixation, thereby promoting plant growth (Thilakarathna et al., 2019; Umar et al., 2020). Nodule volume, count, and weight are increased in our study (Table 5).

Due to the incorporation of C_2_H_4_ and increase of root growth in the formation of nodules, the application of L-methionine at low to moderate concentrations had a positive effect on the nodules (nodule number and dry weight), and these positive effects weakened at higher doses. Some studies have also described the direct application of C_2_H_4_ exogenously released compounds (ethephon) to inhibit the formation of nodules (Grobbelaar et al., 1971; Drennan and Norton, 1972; Goodlass and Smith, 1979). This inhibitory effect is due to the high concentration of C_2_H_4_ used. We reported that ethylene gradually but continuously released L-met as a microbial metabolite to the rhizosphere. In addition, the plants’ response to L-met may be due to the interaction of ethylene released by microorganisms using some endogenous sources.

It was found that inoculation with rhizobia affects the physiological characteristics of chickpea (Tables 6, 7). An increase in leghemoglobin content has been recorded in this study, which may be due to better nodulation of chickpeas that results in higher leghemoglobin content in the nodular tissue. Previous studies have shown that because of better root and nodule development, leghemoglobin content in rhizobia is higher (Sidhu et al., 1967). Likewise, L-methionine has a positive effect on leghemoglobin content because it affects nodule formation. These results are similar to our conclusions.

It can be seen from our research that rhizobium and L-methionine have positive effects on plant nutrients, seed proteins, and photosynthetic pigments. Seeds inoculated with rhizobia can increase nodulation, nitrogen absorption, and seed protein because L-methionine has the function of maintaining protein structure for appropriate division, differentiation, and growth of cells. It gives enough nitrogen and sulfur to fulfill plants’ needs, which may be transformed. It is a polyamine and expands with modification of the molecular structure of hormones, allowing for nitrogen movement between cells and organs (Kakkar et al., 2000; Rudresh et al., 2005). It also acts as a buffer and carbon source. Plants treated with rhizobia and trace elements have high chlorophyll content.

Gwata et al. (2003) that chlorotic plants were not similar to plants with dark green leaves when grown in an N-free environment. It may be because the N in dark green leaves originated from biological fixation. Nitrogen-containing compounds produced because of N_2_ fixation are transferred from nodules as ureides (allantoic acid and allantoin) and transferred to leaves for catabolism (Hildebrandt et al., 2015). It improved chlorophyll synthesis in plants, also indicated by better photosynthesis. The amount of chlorophyll dye indicates the physiological health of plants (Miller et al., 2007). By adding amino acids, better root development can enhance nitrogen fixation, thereby inducing the root surface to rise to absorb nutrients (Hildebrandt et al., 2015; Weiland et al., 2016). L-methionine can also be used as a growth regulator of cytokinins, brassinosteroids, and auxins to promote root germination and help plants absorb more nutrients (Davies, 2004). It ameliorates the homeostasis of endogenous hormones (Colla et al., 2017; Rouphael et al., 2017) and is the optimal compound for growing tips of roots (Davies, 2004). Elevated levels of L-methionine can affect plant hormones giving higher chlorophyll and chloroplasts and cytokinins (Bahari et al., 2013; Anne and Thomas, 2015). The expected requirement to promote the use of L-methionine may be the proximity of plant hormones (for example auxin and cytokinin). Plant hormones and signal compounds can increase photosynthetic activity, thereby increasing yield. Another possible mechanism may be the faster growth of roots treated with L-met. It gives a higher surface area for nutrients and water absorption (Colla et al., 2017; Rouphael et al., 2017). Its effects are then altered to better the cell growth and biomass of plants (Fawzy et al., 2012). It modifies normal growth behavior for good (Bahari et al., 2013; Romero et al., 2014).

## Materials and Methods

### Sampling, Isolation, and Culturing Condition

Chickpea plants with rich pink and healthier nodules were carefully uprooted from different fields of the Ayub Agricultural Research Institute (AARI) in Faisalabad, Pakistan, and transported to the Soil Bacteriology Section of Agri. Biotechnology Research Institute, Faisalabad, Pakistan in plastic bags. Standard procedures (Vincent, 1970) were used to isolate and purify rhizobia from these nodules in the Yeast Extract Mannitol agar media. Purified rhizobia isolates were cultured in Yeast Mannitol Agar slants at 28°C, further kept at 4°C for daily work, and stored at −80°C in 20% (w/v) glycerol for long-time use.

### Study on Genotypic and Phenotypic Characteristics of the Isolates

Fresh culture of each of the ten pure rhizobia isolates was selected for Gram reaction, and then further exposed to various morphological and biochemical tests. The ability of the rhizobia isolates to grow at different salinity levels was studied by culturing yeast extract mannitol (YEM) broth modified with NaCl levels: 0.5, 1, 1.5, 2, and 2.5% (w/v) (Graham et al., 1991). By adjusting the pH value to the range of 4 to 8 in triplicate (Naveed et al., 2014), the potential of these bacterial isolates to grow at various pH levels was studied. A triple iron sugar test of all the isolates was performed using different carbon sources, i.e., glucose, sucrose, and maltose (Hajnaa, 1945), to assess the ability of the isolates to ferment carbohydrates. In addition, other biochemical tests such as fluorescence measurement, urease, citrate starch hydrolysis, Congo red, and bromothymol blue test (Vincent, 1970) were carried out. Selected *Rhizobium* isolate identification and confirmation were performed with the Biolog^®^ system of identification, as given in Table 1 (Microlog System Release 4.2; Biolog Inc., Hayward, CA, United States).

### Biofilm Assay and Exopolysaccharide Production

In accordance with Mirani and Jamil (2011), some modifications were made to analyze the biofilm formation of these bacterial isolates. For quantitative measurement, 10 μl of the broth culture (OD600 = 1) of each isolate was inoculated into a 24-well microplate containing 1 ml of Luria Bertani (LB), and then incubated at 30°C for 96 h. After carefully removing the medium, we rinsed the subsequent wells with sterile water. We stained the biofilm with a crystal violet solution (1%) for 15 min and washed the excess dye with water. The dye (crystal violet) bound to the biofilm structure was extracted with pure ethanol, and the absorbance of contents was taken at 570 nm with a Multiskan Spectrum microplate reader (Thermo Fisher Scientific, Waltham, MA, United States). Qualitative determination of the production of extracellular polysaccharides was carried out according to Paulo et al. (2012).

### Growth Culture and Plant Growth-Enhancing Traits

Bacterial isolates of the chickpea nodules were analyzed for bacterial growth and production of IAA. This test was performed in the YEM medium, which contained mannitol (1%) and L-tryptophan (0.1%) as described by Skerman (1959). Turbidity of culture indicated bacterial growth, which was recorded at 600 nm with a spectrophotometer. According to Sarwar et al. (1992), the supernatant after centrifugation was used for IAA estimation by spectrophotometry using Salkowski reagent (0.5 M FeCl_3_ 1 ml and 35% perchloric acid 50 ml). *Rhizobium* isolates producing IAA in the highest quantities were selected for studies in the next steps. The Pikovskaya agar plate spot method (Pikovskya, 1948) was followed for testing the phosphate solubilization capability of the test isolates.

### Seed Germination Assay

Chickpea seeds were sorted to exclude small and broken seeds. Seeds with a uniform appearance were taken. Their surface was sterilized with NaOCl (5%) followed by washing with plenty of sterilized water. The seeds were then transferred to paper towels soaked in CaSO_4_ (0.5 mM). The setup was stored in a dark room at 25°C for 7 days, and we recorded germination data with the formula:


%Germination=EmergedseedsSeedssownx 100

### Host Specificity Test and Nodulation

After Biolog^®^ identification, the selected isolates were further confirmed to be *Rhizobium* by testing their ability to produce nodules in chickpea plants in a pot study. The surface-sterilized healthy seeds were inoculated with *Rhizobium* culture (CPRH-10, OD.5, containing 10% raw sugar solution and sterilized peat). The seeds were mixed evenly until the appearance of a thin layer of inoculum. The seeds were then spread on a polyethylene sheet and dried overnight in the laboratory. Then, they were sown in clay pots (10 kg of soil) to promote growth. After 40 days, according to the symbiotic efficiency (SE) formula developed by Gibson (1987) and Beck et al. (1993), plants were carefully uprooted from the soil and washed in a bucket of water with gentle shaking. Various parameters of plants such as root length, shoot length, and shoot dry weight were measured:


SE=InoculatedplantdrymatterUninoculatedplantdrymatterx 100

(SE, symbiotic efficiency in %).

### Field Experiment

*Rhizobium* inoculation was tested solely or together with three different L-methionine (L-met) levels (5, 10, and 15 mg L^–1^) on growth and yield parameters of chickpea in a field experiment for two successive years at PulsesResearch Institute, AARI, Faisalabad, Pakistan. The analysis of the soil samples in the test field showed that the soil was sandy clay loam; pH was 7.9; EC was 1.26 dS ml^–1^; the organic matter was 0.78%; total nitrogen was 0.035%; available P was 7.67 mg kg^–1^; extractable K was 115 mg kg^–1^. The experiment included 8 treatments: control, *Rhizobium* inoculation, L-met (5 mg L^–1^), L-met (10 mg L^–1^), L-met (15 mg L^–1^), *Rhizobium* + L-met (5 mg L^–1^), *Rhizobium* + L-met (10 mg L^–1^), and *Rhizobium* + L-met (15 mg L^–1^). The surface-sterilized chickpea seeds are inoculated with selected rhizobia CPRH-10 (based on growth-promoting properties) coated with the aforementioned slurry and soaked using three concentrations of L-methionine, i.e., 5, 10, and 15 mg L^–1^ as per the treatment plan. The experiment was carried out with a randomized complete block design (RCBD) and was repeated three times. One bag of DAP and one bag of sulfate of potash (SOP) were added to the field after the preparation of plots and before sowing. There were 24 plots, and the plot size was 4.8 m^2^ (4 m × 1.2 m). Four rows were maintained in each plot with row × row distance of one foot, and 25–30 seeds per row were used. Commercial chickpea variety Bittal-2016 was selected for sowing based on high yield and disease resistance. The average minimum and maximum temperatures during the crop growth period were 9.1 and 23.7°C, respectively. During the chickpea growing period, rainfall was 25 mm. The research site has a subtropical climatic condition and lies between latitude 31.4504° N and longitude 73.1350° E, with an elevation of about 189 m at sea level. All the needed cultural practices, such as weeding, irrigation, and hoeing, were performed as per the common procedure of the area.

### Agronomic Traits

This study recorded the daily emergence count of above soil surface seedlings with a minimum of 1 cm height and calculated the percentage of emergence as described above. This count took 28 days. Maturity time was measured in days to 70% physiological maturity. Randomly, 5 plants were selected to obtain nodule data by destructive sampling from the border row during the mid-flowering period of chickpea. The excavated root-soil was washed. The nodules were removed from the crown and lateral roots and placed in plastic bags for counting. Total nodule number was counted by considering the pink color of nodules and further analyzed for their volume and dry weight. To record nodule volume, the collected effective nodules were immersed in a measured volume of water in a measuring cylinder. The volume of water displaced by the nodules was considered as nodule volume (cm^3^). After the determination of nodule volume, the collected nodules were dried in an oven for 65 h at 75°C to a constant weight to determine nodule dry weight per plant. The average from the five plants was taken as nodule dry weight per plant. The average value of effective (pink) nodules from the five plants was considered as nodule count per plant. We randomly selected 5 plants from the center row and measured their height with a tape measure, and their average was considered as plant height from each plot. At maturity, a random selection of five plants was considered from harvestable rows of plots for pods per plant. The yield was recorded after the chickpea plants were harvested. We calculated the weight of 100 seeds from each seed batch processed, and then we calculated the average value. Harvest index was calculated from the given formula:


Harvestindex(HI)=SeedyieldPlantbiomassx 100

### Physiological Traits

At noon (between 10:00 and 14:00) after 60 DAS, physiological parameters were recorded from completely green leaves. For chlorophyll a and b content, plant leaves were crushed in acetone and further centrifuged at 1,000 rpm for 10 min. Then, the supernatant was subjected for the recording of the absorbance of chlorophyll “a” at 645 nm and that of “b” at 663 nm, and 480 nm for carotenoid contents using a spectrophotometer (Arnon, 1949). To determine the volume of pink bacteroid tissue of nodules, root nodules were taken at the flowering stage, and a minor section of nodules (5 μl) was made by using sharp. The amount of pink bacteroid tissue comprising leghemoglobin present in the nodule cortex was noted following the method described by Sadasivan and Manickam (2008). The content of IAA in the rhizosphere was tested after 15 and 30 days according to the method given by Sarwar et al. (1992).

### Physicochemical Analysis of Plant and Soil

The soil samples (before and after sowing) were analyzed for contents of N, P, and K. After harvest, seed and straw samples were collected and dried at 65°C for 24 h. The dried samples were ground, and the wet digestion method given by Wolf (1982) was used to determine N and P contents. The protein content of the dried seeds was estimated as per the method given by Lowry et al. (1951).

### Statistical Analysis

Analysis of variance was performed on research data related to growth, yield, and biochemical parameters (Steel et al., 1997). The least significant difference (LSD) test (IBM SPSS Statistics 19, United States) was performed to determine significant differences among the treatment methods.

## Conclusion

In this study, the combined use of *Rhizobium* and 3 levels of L-methionine substantially enhanced the attributes of growth, physiological, chemical contents, and productivity of chickpea in two successive years of field trials. It could meet our interest in validating the benefits of biological inputs and reducing reliance on inorganic fertilizers and destructive farming practices. However, the combined use of *Rhizobium* and L-methionine, specifically a lower dose of L-methionine (5 mg L^–1^) was the most effective in improving the productivity of chickpea. Hence, combined use of L-methionine and *Rhizobium* inoculation may be promoted to produce food using sustainable and safer inputs and preserve the most precious natural resources. Regardless of the various benefits of plant microbiome inoculation and amino acid application, there is a variation in the implementation of such types of practices by the farmer community. Therefore, researchers may now focus on reducing constraints of farmers by developing alternate technologies including integrated use of L-met and rhizobia in farming practices.

## Data Availability Statement

The original contributions presented in the study are included in the article/supplementary material, further inquiries can be directed to the corresponding author/s.

## Author Contributions

MR and AA contributed to conceptualization. MR contributed to methodology and writing (original draft preparation). MR, MB, AAA-H, and AN contributed to software. MN, AAA-H, and AA contributed to validation. MR, AAA-H, MHS, and TA contributed to formal analysis. JH, AAA-H, and MHS contributed to resources. MR and MN contributed to data curation. MN, TA, AAA-H, MHS, and JK contributed to writing (review and editing). MN, AK, JK, MB, and AA contributed to supervision. MN and AM contributed to project administration. AAA-H and AM contributed to funding acquisition. All authors have read and agreed to the published version of the manuscript.

## Conflict of Interest

AK was employed by the company Agricultural Research, Ltd. The remaining authors declare that the research was conducted in the absence of any commercial or financial relationships that could be construed as a potential conflict of interest.

## Publisher’s Note

All claims expressed in this article are solely those of the authors and do not necessarily represent those of their affiliated organizations, or those of the publisher, the editors and the reviewers. Any product that may be evaluated in this article, or claim that may be made by its manufacturer, is not guaranteed or endorsed by the publisher.
